# Inter-pregnancy weight change impacts placental weight and is associated with the risk of adverse pregnancy outcomes in the second pregnancy

**DOI:** 10.1186/1471-2393-14-40

**Published:** 2014-01-22

**Authors:** Jacqueline M Wallace, Sohinee Bhattacharya, Doris M Campbell, Graham W Horgan

**Affiliations:** 1 Lifelong Health Division, Rowett Institute of Nutrition and Health, University of Aberdeen, Aberdeen AB21 9SB, UK; 2 Dugald Baird Centre for Research on Women’s Health, Aberdeen Maternity Hospital, Aberdeen AB25 2ZD, UK; 3 Biomathematics & Statistics Scotland, Aberdeen AB21 9SB, UK

**Keywords:** Inter-pregnancy weight change, Placenta, Pregnancy complications, Birth weight, Parity, Maternal body mass index

## Abstract

**Background:**

The inter-pregnancy period is considered a teachable moment when women are receptive to weight- management guidance aimed at optimising pregnancy outcome in subsequent pregnancies. In population based studies inter-pregnancy weight change is associated with several adverse pregnancy outcomes but the impact on placental size is unknown.

**Methods:**

The association between inter-pregnancy weight change and the primary risk of adverse pregnancy outcomes in the second pregnancy was investigated in 12,740 women with first two consecutive deliveries at a single hospital using logistic regression.

**Results:**

Compared with women who were weight stable, weight loss (>1BMI unit) between pregnancies was associated with an increased risk of spontaneous preterm delivery, low placental weight and small for gestational age (SGA) birth, while weight gain (>3BMI units) increased the risk of pre-eclampsia, gestational hypertension, emergency caesarean section, placental oversize and large for gestational age (LGA) birth at the second pregnancy. The relationship between weight gain and pre-eclampsia risk was evident in women who were overweight at first pregnancy only (BMI ≥25 units), while that between weight loss and preterm delivery was confined to women with a healthy weight at first pregnancy (BMI <25 units). In contrast, the association between weight loss and SGA was independent of first pregnancy BMI. A higher percentage of women who were obese at first pregnancy were likely to experience a large weight gain (P < 0.01) or weight loss (P < 0.001) between consecutive pregnancies compared with the normal BMI reference group.

**Conclusion:**

Inter-pregnancy weight change in either direction increases the risk of a number of contrasting pregnancy complications, including extremes of placental weight. The placenta may lie on the causal pathway between BMI change and the risk of LGA or SGA birth.

## Background

Both pre-pregnancy maternal underweight and obesity are associated with a myriad of adverse pregnancy outcomes for both mother and child, which impact their health and wellbeing, and draw substantially on limited health service resources [1-3]. Women who are underweight at conception are at risk of fetal growth restriction, premature delivery and low birth weight [4-7]. At the other end of the BMI spectrum, maternal obesity is commonly associated with a plethora of risks that generally increase with degree of overweight including hypertensive disorders, gestational diabetes, stillbirth, induction of labour, caesarean delivery, large for gestational age (LGA, birth weight >90^th^ after adjustment for gender and gestational age) and preterm birth [4,8-11]. Avoiding the consequences of these BMI extremes by encouraging women to achieve a healthy BMI prior to their first pregnancy is a laudable goal but it is pertinent to recognise that many of these pregnancies are largely unplanned. This is particularly true for younger and socially deprived women who predominate in the underweight and obese BMI categories, respectively [12-14]. The inter-pregnancy period may be a “teachable moment” when women are receptive to guidance to achieve or maintain a healthy weight and thereby optimise pregnancy outcome in their second pregnancy. This guidance must be evidence based and applicable to all women across the BMI range. Previous population based studies from Sweden and the USA (~114-230,000 women) have examined inter-pregnancy BMI changes in relation to a number of pregnancy complications [15-22]. Using contrasting approaches these researchers have demonstrated a strong relationship between inter-pregnancy weight gain and the risk of pre-eclampsia, stillbirth, maternal diabetes, LGA and caesarean delivery. Villamer & Cnattingius [15] additionally detected a linear relationship between inter-pregnancy weight gain and the incidence of gestational hypertension while Whiteman et al. [22] reported that inter-pregnancy BMI loss resulting in a shift from the normal to underweight category increased the risk of spontaneous preterm delivery. The effect of inter-pregnancy BMI change on the primary incidence of small for gestation age (SGA) has not been documented.

We recently demonstrated that placental growth as reflected by weight at delivery is an independent risk factor for the range of pregnancy complications shown to be influenced by the contrasting ends of the BMI spectrum in the pregnant population irrespective of parity [23]. Inter-pregnancy BMI change may also influence placental weight at the second pregnancy and hence hypothetically the placenta may lie in the causative pathway between BMI change and the risk of specific adverse pregnancy outcomes. The aims herein were three-fold. Firstly we aimed to determine if both extremes of inter-pregnancy weight change influenced the risk of a range of pregnancy outcomes, including SGA births, in a relatively small cohort of women delivering at a single maternity unit in the UK. We deliberately used the same analytical approach as Villamer and Cnattingius [15] to facilitate comparison of common elements of our data. Secondly we examined whether the relationships between inter-pregnancy weight change and the primary incidence of pregnancy complications at second pregnancy differed in women who had a healthy or unhealthy BMI at the first pregnancy. Thirdly, we investigated whether inter-pregnancy BMI change influenced the incidence of low placental weight (<10^th^ centile after adjustment for gender and gestational age) or placental oversize (>90^th^ centile after adjustment) at the second pregnancy.

## Methods

### Study population

This was a retrospective cohort study using data from the Aberdeen Maternity and Neonatal Databank (AMND). Ethical approval was granted by the North of Scotland Research Ethics Service (REC Ref 13/NS/0050) for all observational studies using routinely collected anonymised data from the AMND, provided permission was granted by the Steering Committee (Caldicott guardians). After obtaining permission from the Steering Committee of the AMND, routinely collected anonymised data were extracted from the databank for all singleton births after 24 weeks’ gestation in Aberdeen city and district between 1986 and 2007. The study population of interest was women who had their first-ever and second consecutive births at Aberdeen Maternity Hospital, who booked before 24 weeks gestation on both occasions and whose height and weight was recorded. After excluding women (n = 955) with missing or improbable data for key variables (placental weight, birth weight or baby gender), a final study population of 12,740 women were available for analysis.

### Study design

Maternal weight at the first antenatal visit for each pregnancy was adjusted to take into account stage of gestation when weight was measured exactly as described previously using maternal conformation data for women from the same geographical area [24]. Briefly this involved obtaining the z score of weight for height (difference between weight and mean weight for height, relative to standard deviation), adjusting this by adding a constant that depended on stage of gestation, and then recalculating a weight only from this adjusted z score. The resulting corrected weight was then used together with the unadjusted height to calculate an adjusted BMI (weight/height^2^). Although the mean individual difference in gestational age at the initial hospital visit in the first versus second pregnancy was small (1 ± 3 weeks, mean ± SD) this approach meant that the maternal BMI calculated for both pregnancies was corrected to a standard stage of gestation for all women in the study population. Moreover as a small percentage of women had their first hospital visit after 16 weeks gestation (13 and 7% of women in first and second pregnancies, respectively) this approach facilitated the inclusion of women who had a first hospital booking appointment up to 23 weeks gestation. These adjusted maternal BMIs were used to calculate the inter-pregnancy change in BMI. The difference was categorised as less than −1 (BMI loss of greater than 1 unit), -1 to less than 1 (BMI stable = reference group), 1 to less than 3 (modest gain) and 3 or more units (large gain) and replicate the BMI categories used in the Scandinavian population based study reported by Villamor and Cnattingius (15). Initial BMI at the beginning of the first pregnancy was categorised using conventional cut-off points for underweight (<18.5), normal weight (18.5-24.9), overweight (25.0-29.9) or obese (≥30). Other covariates including maternal age, height, year of delivery and smoking habit were grouped as detailed in Table 1. The inter-delivery interval was calculated in years between the birth of the first and second child.

**Table 1:** Maternal characteristics and first pregnancy outcome in relation to change in BMI between first and second pregnancy

		**BMI change category (number of women)**						
**Maternal characteristics and/or complications during first pregnancy**		<−1*	−1 to <1*	1 to <3*	≥3*	**P value**^**¥**^	**Mean change in BMI (SD)**	**P value**
		**(n = 1637)**	**(n = 5586)**	**(n = 3740)**	**(n = 1777)**			
Age (years)								
≤19		11.1	32.7	28.4	27.8	P = 0.000	1.860 (2.961)	P = 0.000
20-24		14.3	39.8	29.4	16.5		1.122 (2.392)	
25-29		12.8	47.1	29.8	10.3		0.790 (1.884)	
30-34		11.8	51.1	29.1	8.0		0.696 (1.726)	
≥35		13.5	49.3	29.3	7.9		0.615 (1.743)	
Height (cm)								
≤159		12.7	41.9	30.3	15.1	P = 0.015	1.098 (2.283)	P = 0.002
160-164		12.6	44.6	28.8	14.0		0.992 (2.235)	
165-169		12.8	44.6	29.2	13.4		0.973 (2.136)	
≥170		13.8	45.9	28.6	11.7		0.870 (2.101)	
Adjusted BMI (kg/m^2^)								
≤18.5		2.6	47.2	37.9	12.3	P = 0.000	1.396 (1.757)	P = 0.000
18.6-24.9		9.8	49.7	29.7	10.8		0.920 (1.853)	
25-29.9		18.3	35.5	29.1	17.1		1.069 (2.525)	
≥30		20.7	26.4	25.7	27.2		1.361 (3.334)	
Smoking habit								
Non-smoker		12.2	45.0	29.7	13.1	P = 0.000	0.991 (2.161)	P = 0.267
Smoker		15.0	40.3	28.0	16.7		1.057 (2.380)	
Inter-delivery interval (years)								
≤1		17.5	48.6	26.4	7.5	P = 0.000	0.508 (1.854)	P = 0.000
2		15.6	51.5	25.0	7.9		0.503 (1.823)	
3		12.3	46.6	30.2	10.9		0.871 (1.938)	
>3		9.2	33.0	33.7	24.1		1.747 (2.615)	
Pre-eclampsia	No	12.8	44.0	29.4	13.8	P = 0.029	0.999 (2.204)	P = 0.080
	Yes	13.7	40.5	28.0	17.8		1.160 (2.381)	
Gestational hypertension	No	13.1	45.2	28.4	13.3	P = 0.000	0.959 (2.199)	P = 0.000
	Yes	12.1	38.7	32.9	16.3		1.189 (2.259)	
Induced labour	No	12.8	44.9	29.0	13.3	P = 0.000	0.968 (2.170)	P = 0.001
	Yes	13.1	41.2	30.2	15.5		1.107 (2.317)	
Instrumental delivery	No	13.0	44.1	28.3	14.6	P = 0.000	1.017 (2.263)	P = 0.431
	Yes	12.4	43.2	32.0	12.4		0.982 (2.076)	
Elective caesarean	No	12.8	43.8	29.4	14.0	P = 0.733	1.011 (2.217)	P = 0.340
	Yes	11.9	46.5	28.1	13.5		0.905 (2.121)	
Emergency caesarean	No	12.7	44.2	29.2	13.9	P = 0.089	1.012 (2.209)	P = 0.485
	Yes	14.2	41.2	30.5	14.1		0.972 (2.242)	
Spontaneous preterm	No	13.0	43.8	29.4	13.8	P = 0.254	1.004 (2.223)	P = 0.614
	Yes	10.8	45.0	29.3	14.9		1.044 (2.080)	
Post-term delivery	No	12.9	43.9	29.3	13.9	P = 0.507	1.000 (2.211)	P = 0.180
	Yes	11.7	42.6	31.3	14.4		1.109 (2.245)	
Placental abruption	No	12.9	44.0	29.2	13.9	P = 0.005	1.004 (2.218)	P = 0.213
	Yes	10.4	34.4	40.1	15.1		1.205 (1.906)	
Placenta praevia	No	12.9	43.9	29.3	13.9	P = 0.701	1.007 (2.214)	P = 0.981
	Yes	4.5	50.0	31.8	13.7		1.019 (2.016)	
Postpartum haemorrhage	No	12.9	43.9	29.4	13.8	P = 0.247	0.998 (2.211)	P = 0.148
	Yes	12.0	43.0	29.3	15.7		1.093 (2.230)	
Stillbirth	No	12.9	43.8	29.3	14.0	P = 0.300	1.007 (2.218)	P = 0.807
	Yes	8.4	48.6	32.7	10.3		0.955 (1.619)	
Birth weight <10^th^ C (SGA)	No	13.0	43.5	29.6	13.9	P = 0.134	1.014 (2.223)	P = 0.331
	Yes	11.8	46.6	27.6	14.0		0.953 (2.134)	
Birth weight >90^th^ C LGA)	No	12.8	44.1	29.4	13.7	P = 0.014	0.994 (2.201)	P = 0.023
	Yes	13.4	40.4	29.1	17.1		1.161 (2.346)	
Placental weight <10^th^ C	No	12.9	43.4	29.4	14.3	P = 0.001	1.023 (2.229)	P = 0.012
	Yes	12.1	48.3	28.8	10.8		0.855 (2.055)	
Placental weight >90^th^ C	No	12.7	44.5	29.1	13.7	P = 0.001	0.989 (2.190)	P = 0.006
	Yes	13.7	38.8	31.2	16.3		1.163 (2.396)	

*Values are percentage of women. ^¥^P values obtained from a chi-squared test of counts.

The pregnancy complications and obstetric outcomes assessed included pre-eclampsia and gestational hypertension (coded according to the ISSHP definition, [25]), placental abruption, placenta praevia, postpartum haemorrhage (defined as blood loss of more than 500 ml or 1000 ml at vaginal or Caesarean delivery respectively), type of labour (spontaneous or induced), type of delivery (spontaneous vaginal, instrumental, elective or emergency Caesarean section), spontaneous preterm delivery (<37weeks) and post-term delivery (>41 weeks). Gestational age was recorded according to the last menstrual period and was confirmed by ultrasound. Where there was disagreement of more than 7 days, the ultrasound date was taken as the actual gestational age. Perinatal outcomes included stillbirth, birth weight and placental weight. The latter was weighed untrimmed and recorded to the nearest 10 g. Birth weight was defined as small for gestational age (SGA) if weight was less than the 10^th^ centile or large for gestational age (LGA) if weight was above the 90^th^ centile for gestation using gender and parity specific birth weight charts for Scottish singleton births [26]. Similarly gestational age, gender and parity grouping specific placental weight charts were used to define low placental weight (<10^th^ centile) and placental oversize (>90^th^ centile). These were derived from the AMND [27].

### Statistical analysis

In Table 1 the frequency of maternal characteristics and outcomes of first pregnancy in relation to the BMI change category between pregnancies was analysed by Chi-Square tests. In addition the distribution of inter-pregnancy weight (BMI) change as a continuous variable was compared with maternal characteristics and outcomes of the first pregnancy as categorical predictors using one-way ANOVA. The effect of initial BMI category at first pregnancy and direction of BMI change category between pregnancies on inter-pregnancy BMI change was analysed using ANOVA (Linear Model) and differences between BMI groups were compared by Tukey’s method (data in text). The significance of any trend in the incidence rate of each pregnancy complication in the second pregnancy in relation to the BMI change since the first pregnancy (Table 2) was evaluated by the Cochran-Armitage test for (a) women who experienced the specific complication in the second pregnancy only and (b) for all women irrespective of first pregnancy history. The latter may be of value in estimating trends and risk in situations where the first pregnancy complications have not been clinically documented. The risk of pregnancy complications in the second pregnancy in relation to inter-pregnancy BMI change were assessed using logistic regression (Table 2). Risks are presented as Odds Ratios (OR) with 95% confidence intervals (CI) and were adjusted for BMI at first pregnancy, height, inter-delivery interval, along with maternal age and smoking status, year of delivery, baby gender and gestational age at second pregnancy. Where appropriate, variables were additionally adjusted for the co-occurrence of either pre-eclampsia or gestational hypertension. After testing for an interaction between BMI category at baseline and BMI change category, we further investigated whether BMI at the beginning of the first pregnancy modified any relationship between inter-pregnancy BMI change and pregnancy complication risk at the second pregnancy by repeating the above logistic regression approach for women who had a BMI below or above 25 at the first pregnancy (Table 3).

**Table 2:** Frequency rate and adjusted odds ratios for adverse perinatal outcomes during second pregnancy in relation to change in BMI from first pregnancy (a) for women with specific complication in second pregnancy only and (b) for all women irrespective of first pregnancy history

		**BMI change (% study population)**				
**(a) Complication 2**^**nd **^**pregnancy only (cases)**		**<−1†**	**−1 to <1**	**1 to <3**	**≥3**	**Trend**^**¥**^**, P value**
		**(13%)**	**(44%)**	**(29%)**	**(14%)**	
Pre-eclampsia	Rate (%)	1.11	0.74	1.22	2.07	<0.001
(n = 140)	OR (95% CI)	1.23 (0.69-2.20)	1	1.44 (0.93-2.23)	1.85 (1.12-3.04)*	0.100
Gestational	Rate (%)	3.54	3.55	4.47	6.98	<0. 001
Hypertension (n = 524)	OR (95% CI)	0.83 (0.61-1.14)	1	1.22 (0.98-1.52)	1.82 (1.40-2.36)***	<0.001
Induced labour	Rate (%)	13.13	10.70	12.55	14.42	0.032
(n = 1371)	OR (95% CI)	1.21 (1.01-1.46)*	1	1.12 (0.98-1.29)	1.17 (0.97-1.40)	0.100
Elective caesarean	Rate (%)	7.34	7.01	8.56	9.69	0.001
(n = 930)	OR (95% CI)	0.91 (0.72-1.15)	1	1.16 (0.98-1.37)	1.18 (0.95-1.48)	0.093
Emergency caesarean	Rate (%)	3.74	2.72	3.92	6.79	<0. 001
(n = 461)	OR (95% CI)	1.27 (0.93-1.75)	1	1.30 (1.03-1.66)*	1.78 (1.35-2.35)***	<0.001
Spontaneous preterm	Rate (%)	5.89	4.39	4.53	4.1	0.072
(<37 wks, n = 558)	OR (95% CI)	1.46 (1.08-1.97)*	1	1.00 (0.78-1.28)	0.65 (0.45-0.93)*	0.002
SGA, <10^th^ C	Rate (%)	9.57	6.42	6.31	5.83	<0.001
(n = 800)	OR (95% CI)	1.65 (1.33-2.04)***	1	0.95 (0.80-1.14)	0.82 (0.63-1.05)	<0.001
LGA, >90^th^ C (n = 755)	Rate (%)	3.48	5.22	7.78	9.42	<0. 001
	OR (95% CI)	0.57 (0.42-0.76)***	1	1.48 (1.24-1.76)***	1.70 (1.36-2.13)***	<0.001
Placental wt. <10thC	Rate (%)	8.25	6.06	5.45	5.34	<0. 001
(n = 773)	OR (95% CI)	1.67 (1.35-2.07)***	1	0.91 (0.76-1.09)	0.99 (0.77-1.28)	<0.001
Placental wt. >90thC	Rate (%)	7.33	6.96	8.93	12.27	<0. 001
(n = 1061)	OR (95% CI)	0.90 (0.72-1.11)	1	1.26 (1.08-1.47)**	1.59 (1.31-1.47)***	<0.001
**(b) Complication all women (cases)**						
Pre-eclampsia	Rate (%)	1.68	1.27	1.93	3.31	<0. 001
(n = 225)	OR (95% CI)	0.97 (0.60-1.55)	1	1.33 (0.94-1.88)	1.70 (1.14-2.53)**	0.033
Gestational hypertension (n = 967)	Rate (%)	7.06	6.4	9.74	12.04	<0. 001
	OR (95% CI)	0.90 (0.72-1.14)	1	1.50 (1.28-1.77)***	1.79 (1.46-2.20)***	<0.001
Induced labour	Rate (%)	23.27	20.1	24.45	29.71	<0. 001
(n = 2386)	OR (95% CI)	1.07 (0.92-1.24)	1	1.13 (1.01-1.26)*	1.20 (1.03-1.39)*	0.057
Elective caesarean	Rate (%)	9.06	8.68	10.06	11.48	0.004
(n = 1107)	OR (95% CI)	0.91 (0.74-1.13)	1	1.10 (0.94-1.29)	1.15 (0.93-1.41)	>0.1
Emergency caesarean	Rate (%)	7.69	6.24	7.74	10.65	<0. 001
(n = 885)	OR (95% CI)	1.08 (0.86-1.36)	1	1.12 (0.94-1.33)	1.24 (1.00-1.54)*	>0.1
Spontaneous preterm (<37 wks, n = 745)	Rate (%)	7.41	6.13	5.89	6.03	>0.1
	OR (95% CI)	1.23 (0.90-1.67)	1	0.91 (0.71-1.16)	0.71 (0.50-1.01)	0.057
SGA, <10^th^ C (n = 1203)	Rate (%)	14.55	10.52	9.29	8.88	<0. 001
	OR (95% CI)	1.63 (1.36-1.95)***	1	0.85 (0.74-0.99)*	0.78 (0.63-0.97)*	<0.001
LGA, >90^th^ C	Rate (%)	6.09	7.71	11.11	14.49	<0. 001
(n = 1093)	OR (95% CI)	0.64 (0.50-0.82)***	1	1.44 (1.23-1.67)***	1.82 (1.50-2.20)***	<0.001
Placental wt. <10thC	Rate (%)	9.95	8.3	6.89	6.41	<0. 001
(n = 999)	OR (95% CI)	1.53 (1.26-1.87)	1	0.84 (0.71-0.98)*	0.88 (0.70-1.11)	<0.001
Placental wt. >90thC	Rate (%)	9.89	9.47	11.87	16.54	<0. 001
(n = 1429)	OR (95% CI)	0.84 (0.69-1.01)	1	1.24 (1.08-1.42)**	1.59 (1.34-1.89)***	<0.001

Odds ratios (OR) and 95% confidence limits (CI) from logistic regression *P < 0.05, **P < 0.01 ***P < 0.001 relative to stable BMI reference group (OR = 1). Models adjusted for baseline BMI at first pregnancy, height and inter-delivery interval, and maternal age, year of delivery and smoking status, gestational age and baby gender at second pregnancy Where appropriate, variables were additionally adjusted for the co-occurrence of pre-eclampsia or gestational hypertension. For (a), women with each individual complication in the first pregnancy were excluded. † Refers to BMI loss between pregnancies of more than 1 unit. ^¥^Significance of trend for complication frequency rate from the Cochran –Armitage test and for odds ratios from calculation of overall Chi square value. *SGA*, Small for gestational age; *LGA*, Large for gestational age. Birth and placental weight categories based on gender and parity specific centile charts for Scotland [26,27].

**Table 3:** Frequency rate and adjusted odds ratios for adverse perinatal outcomes during second pregnancy in relation to change in BMI from first pregnancy for women with BMI (a) less than 25 and (b) more than 25 units in first pregnancy

		**BMI change**				
**(a) First pregnancy BMI <25, complication 2**^**nd **^**pregnancy (cases)**		**<−1†**	**−1 to <1**	**1 to <3**	**≥3**	**Trend**^**¥**^**, P value**
Pre-eclampsia	Rate (%)	1.02	0.76	0.98	1.35	>0.1
(n = 75)	OR (95% CI)	1.43 (0.64-3.20)	1	1.14 (0.66-1.98)	1.27 (0.60-2.68)	>0.1
Gestational hypertension (n = 282)	Rate (%)	2.86	3.04	3.86	5.52	**<0.001**
	OR (95% CI)	0.87 (0.54-1.39)	1	1.30 (0.99-1.73)	1.98 (1.35-2.90)***	0.002
Emergency caesarean	Rate (%)	3.67	2.24	3.43	8.06	<0. 001
(n = 267)	OR (95% CI)	1.73 (1.11-2.69)*	1	1.41 (1.03-1.92)*	2.64 (1.82-3.81)***	<0.001
Spontaneous preterm (<37 wks, n = 402)	Rate (%)	7.47	4.78	5.05	4.78	>0.1
	OR (95% CI)	1.89 (1.30-2.74)***	1	0.62 (0.38-1.02)	0.62 (0.38-1.02)	<0.001
SGA, <10^th^ C	Rate (%)	12.36	7.33	7.05	6.52	0.001
(n = 585)	OR (95% CI)	1.76 (1.35-2.28)***	1	0.90 (0.74-1.11)	0.69 (0.50-0.96)*	<0.001
LGA, >90^th^ C	Rate (%)	1.80	3.90	6.96	6.15	<0. 001
(n = 381)	OR (95% CI)	0.44 (0.25-0.76)**	1	1.84 (1.46-2.32)***	1.83 (1.28-2.60)***	<0.001
Placental wt. <10thC	Rate (%)	11.40	7.24	6.54	6.90	0.005
(n = 568)	OR (95% CI)	1.79 (1.36-2.34)***	1	0.92 (0.74-1.13)	1.05 (0.76-1.44)	<0.001
Placental wt. >90thC	Rate (%)	4.49	5.94	9.08	11.69	<0. 001
(n = 564)	OR (95% CI)	0.69 (0.47-1.00)*	1	1.49 (1.22-1.82)***	1.76 (1.33-2.32)***	<0.001
**(b) First pregnancy BMI >25, complication 2**^**nd**^** pregnancy (cases)**						
Pre-eclampsia	Rate (%)	1.20	0.68	1.69	2.81	<0.001
(n = 65)	OR (95% CI)	1.47 (0.60-3.63)	1	2.29 (1.06-4.95)*	3.03 (1.38-6.64)**	0.029
Gestational hypertension (n = 242)	Rate (%)	4.19	5.02	5.68	8.52	<0.001
	OR (95% CI)	0.76 (0.50-1.17)	1	1.09 (0.77-1.54)	1.64 (1.14-2.36)**	0.004
Emergency caesarean	Rate (%)	3.80	4.07	4.89	5.52	0.063
(n = 194)	OR (95% CI)	0.88 (0.56-1.38)	1	1.14 (0.78-1.65)	1.11 (0.73-1.69)	>0.1
Spontaneous preterm (<37 wks, n = 156)	Rate (%)	4.44	3.34	3.52	3.41	>0.1
	OR (95% CI)	1.01 (0.60-1.69)	1	0.97 (0.60-1.56)	0.67 (0.38-1.19)	>0.1
SGA, <10^th^ C	Rate (%)	7.09	3.99	4.89	5.14	>0.1
(n = 215)	OR (95% CI)	1.73 (1.18-2.54)**	1	1.18 (0.81-1.72)	1.13 (0.74-1.73)	0.036
LGA, >90^th^ C	Rate (%)	5.09	9.04	9.43	12.98	<0. 001
(n = 374)	OR (95% CI)	0.55 (0.38-0.79)***	1	1.05 (0.80-1.38)	1.45 (1.08-1.95)*	<0.001
Placental wt. <10thC	Rate (%)	6.81	4.36	4.28	4.39	0.049
(n = 205)	OR (95% CI)	1.56 (1.07-2.29)*	1	0.91 (0.62-1.33)	0.93 (0.60-1.44)	0.026
Placental wt. >90thC	Rate (%)	11.31	12	11.25	16.42	0.023
(n = 497)	OR (95% CI)	0.91 (0.69-1.20)	1	0.94 (0.73-1.20)	1.34 (1.03-1.75)*	0.029

Odds ratios (OR) and 95% confidence limits (CI) from logistic regression *P < 0.05, **P < 0.01 ***P < 0.001 relative to stable BMI reference group (OR = 1). Models adjusted for baseline BMI at first pregnancy, height and inter-delivery interval, and maternal age, year of delivery and smoking status, gestational age and baby gender at second pregnancy. Where appropriate, variables were additionally adjusted for the co-occurrence of pre-eclampsia or gestational hypertension. For each individual endpoint women experiencing the complication in the first pregnancy were excluded. For the < −1, -1 to 1, 1 to 3 and ≥3 BMI change categories the distribution of the study population was 9, 50, 30 and 11% for women with a first pregnancy BMI of less than 25 (a) and 19, 33, 28 and 20% for women with a first pregnancy BMI of more than 25 (b). † Refers to BMI loss between pregnancies of more than 1 unit. ^¥^Significance of trend for complication frequency rate from the Cochran –Armitage test and for odds ratios from calculation of overall Chi square value. *SGA*, Small for gestational age; *LGA*, Large for gestational age. Birth and placental weight categories based on gender and parity specific centile charts for Scotland [26,27].

The Cochran-Armitage test was carried out using R (Foundation for Statistical Computing, Vienna, Austria) and all other statistical analysis was carried out using Minitab (version 15; Minitab Inc., State College, PA).

## Results

### Pregnancy complications and baseline BMI

Maternal and pregnancy outcome characteristics at the first and second pregnancy in relation to BMI category at first pregnancy are detailed (Additional file 1). At the first baseline pregnancy 2% of women were underweight, 63% were normal weight, 26% were overweight and 9% were obese and there was no major difference in inter-delivery interval between groups.

### Pregnancy complications and inter-pregnancy change in BMI

**Figure 1:**
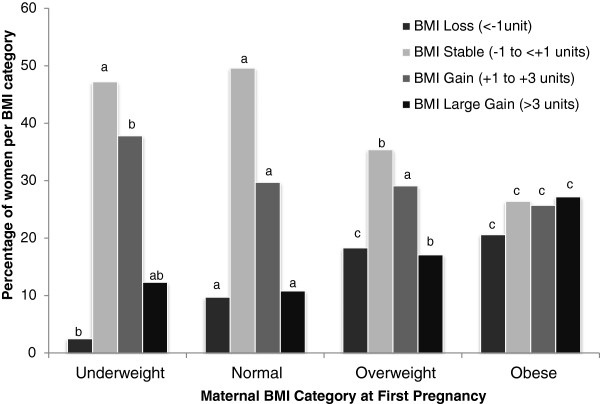
**Percentage of women who were weight stable or who lost or gained weight between pregnancies in relation to maternal BMI category at first pregnancy.** Where superscript letters differ within inter-pregnancy weight change categories, P < 0.01.

On average women gained one BMI unit (median 0.7, IQR −0.3 to 2.0) during a mean pregnancy interval of 3.4 years (median 3.0, IQR 2 to 4). Table 1 details the frequency of maternal characteristics and outcomes of the first pregnancy in relation to the BMI change category between pregnancies. It also details the average change in BMI between the first and second pregnancy in relation to these parameters. The number of women per BMI change category was influenced by age, height, first pregnancy BMI, smoking habit and inter-delivery interval and hence all these parameters were adjusted for in the logistic regression analysis. Similarly, the incidence of pre-eclampsia, gestational hypertension, induced and instrumental deliveries, placental abruption, LGA and extremes of placental weight at the first pregnancy varied by BMI change category. Irrespective of BMI change category, on average, weight gain between the first and second pregnancy was not influenced by smoking habit, decreased with age and height, and increased with the inter-delivery interval. Weight gain between the first and second pregnancy was greater in women who had gestational hypertension, induced labour, LGA and placental weight > 90^th^ centile, and lower in women with a low placental weight in the first pregnancy. The groups of women classified as underweight or obese at baseline had the largest change in average weight between pregnancies (Table 1) and the direction of weight/BMI change is presented in more detail in Figure 1. Of the group of women who were underweight at baseline, few subsequently lost weight and more gained a modest amount of weight compared with all other baseline BMI categories (P < 0.01). While fewer women who were obese at baseline were weight stable or gained a modest amount of weight, more gained a large amount of weight between pregnancies than all other categories (P < 0.01). Counter-intuitively, more initially overweight or obese women also lost weight between pregnancies than in the normal weight stable reference group (P < 0.001). In the relatively weight stable and modest gain groups the extent of individual weight change was independent of initial BMI category. In contrast the extent of inter-pregnancy weight change in the loss group was linearly associated with initial BMI category (equivalent to an average of −1.4, -1.6, -2.1 and −3.0 BMI units for underweight, normal, overweight and obese groups, respectively) and was statistically higher in women initially categorized as obese relative to all other groups (P < 0.001). Similarly for women with a large inter-pregnancy gain in BMI (equivalent to an average of 5.0, 4.7, 5.1 and 5.3 BMI units for underweight, normal, overweight and obese groups, respectively), the gain was highest in the overweight and obese compared with the normal reference group (P < 0.001).

In Table 2 incidence rate and adjusted odds ratios for adverse perinatal outcomes during the second pregnancy, in relation to BMI change category between the first and second pregnancy, are presented for women experiencing the individual complication in the second pregnancy only and for all women irrespective of previous history. In both cases data are shown for those adverse outcomes where weight change was associated with statistically significant linear trends across the BMI change spectrum and/or adjusted odds ratios only (non-significant adjusted odds ratios for the remaining pregnancy complications and unadjusted odds ratios for all pregnancy complications for women experiencing the complication in the second pregnancy only are available in Additional file 2). For women experiencing a complication in the second pregnancy only (Table 2a), a weight gain equivalent to more than 3 BMI units (median 4.3, IQR 3.5 to 5.7) was associated with an increased risk of pre-eclampsia, gestational hypertension, emergency caesarean section and LGA (OR 1.59 to 1.85) and was protective against spontaneous preterm labour (OR 0.65). In contrast women who lost more than 1 BMI unit (median −1.6, IQR −2.3 to −1.3) had a higher risk of induced labour, spontaneous preterm delivery and SGA (OR 1.21 to 1.67) and a lower risk of LGA (OR 0.57) relative to the stable BMI reference group (median 0.1, IQR −0.3 to 0.5). The size and direction of the associations between inter-pregnancy weight change and most pregnancy complications was similar for all women irrespective of first pregnancy history (Table 2b). The exception was the risk of induced labour which was slightly higher in both weight gain categories when all women were included.

### Pregnancy complications, baseline BMI category and inter-pregnancy change in BMI

When the putative interaction between baseline BMI category and inter-pregnancy change in BMI category were examined, statistically significant interactions (weight change effects differing between the four baseline BMI categories) were evident for the risk of some of the most prevalent adverse pregnancy outcomes, namely emergency caesarean section (P = 0.012), LGA (P = 0.009) and placental weight >90^th^ centile (P = 0.004). On this basis and cognizant that this initial approach may miss detecting interactions for less prevalent outcomes we further sub-divided the data and compared the effects of weight change between pregnancies on second pregnancy primary complication risk for women who had a BMI less than 25 (median 22.4, IQR 21.0 to 23.6) with those who had a BMI above 25 (median 27.5, IQR 26.0 to 30.0) at first pregnancy. This equated to 65 versus 35% of the study population, respectively (Table 3). Data are shown for those adverse outcomes where weight change was associated with significant linear trends across the BMI change spectrum and/or adjusted odds ratios in at least one baseline BMI category. Women who were overweight at baseline and who had a modest (median 1.9, IQR 1.4 to 2.4) or large (median 4.5, IQR 3.7 to 6.0) gain in BMI between pregnancies had a two and three fold higher risk of pre-eclampsia at the second pregnancy compared with overweight women who were BMI stable (median 0.1, IQR −0.4 to 0.5). No such relationship was evident in women with baseline BMI less than 25 units (healthy weight). In contrast an 80% greater risk of spontaneous preterm labour was evident in women who lost weight between pregnancies and who had a baseline BMI less than 25 units but not in women who were overweight at baseline. Similarly, both inter-pregnancy weight loss and weight gain heightened the risk of requiring an emergency section in women who had a baseline BMI less than 25 only, with the highest overall risk in the largest BMI gain category. The direction of the association between weight change and the remaining adverse outcomes shown, namely gestational hypertension and both birth weight extremes was similar in both baseline BMI categories but significantly stronger in women whose BMI was less than 25 at the outset. There was no effect of inter-pregnancy weight change on the crude or adjusted risk of stillbirth, post-term delivery, postpartum haemorrhage, placental abruption or placenta praevia (see Additional file 2) in the population overall and when examined in relation to baseline BMI category.

### Inter-pregnancy BMI change and placental weight

Average placental weight increased by 36 g between the first (median 600, IQR 520 to 700 g) and second pregnancy (median 630, IQR 550 to 725 g, P < 0. 001). The difference in placental weight at delivery in successive pregnancies after adjustment for inter-delivery interval and maternal height along with age, gestation length and smoking status in both pregnancies was −0.4, 17.4, 24.1 and 30.2 g for women in the weight loss, weight stable, modest and large weight gain categories, respectively (P < 0.001). In the second pregnancy, inter-pregnancy BMI change, through the BMI loss to large gain categories was inversely related to the incidence of low placental weight (<10^th^ centile) and positively related to the incidence of high placental weight (>90^th^ centile) in the population as a whole (Table 2), and in women who were either overweight or had a relatively healthy weight at baseline (Table 3). Thus for the population overall, women who lost weight between pregnancies (13%) were more likely to have a very small placenta (OR 1.67) while women who gained a modest (29%) or large amount of weight (14%) were more likely to have a large placenta (OR 1.26 and 1.59, respectively).

## Discussion

This study demonstrates that weight change during the inter-pregnancy interval is strongly associated with the risk of experiencing a range of pregnancy complications in the second pregnancy. This was true for women experiencing particular complications for the first time and for all women irrespective of their previous first pregnancy history. The data herein for women encountering a complication for the first time in the second pregnancy can be directly compared with the previous ten-fold larger population based study of Villamor & Cnattingius [15]. For women who gained 3 or more BMI units, this comparison reveals a striking similarity between studies in the adjusted odds ratios for major pregnancy complications including pre-eclampsia (1.85 vs 1.78), gestational hypertension (1.82 vs 1.76), and LGA (1.70 vs 1.87), and a comparable OR for emergency caesarean delivery (present study) compared with all caesareans combined (1.78 vs 1.32). In contrast to previous studies [15,20], inter-pregnancy weight change was not associated with the risk of stillbirth but our inability to detect a relationship may partly be due to a combination of the generally low incidence rate of this complication and the considerably smaller size of our study population. In the present study, inter-pregnancy weight loss was associated with a 46% higher risk of spontaneous preterm delivery while a large BMI gain was modestly protective (35% lower risk). Both the direction and magnitude of these effects were very similar to those reported previously [22], albeit examining BMI changes in a different way and in a large study population of more than 200,000 women. In the latter study which used self-reported weight and height data, moving from a normal to an underweight BMI classification between pregnancies was associated with a 50% higher risk of spontaneous preterm delivery while moving from normal to overweight or obese categories was associated with a 20% lower risk of an early birth in both cases. In addition to confirming the general robustness of these previous observations, herein for the first time we demonstrate that inter-pregnancy weight loss is associated with a 65% higher risk of an SGA birth. Although not directly comparable, weight loss before pregnancy, calculated as an annual average between 20 years of age and start of pregnancy approximately 9 years later, has recently been shown to increase the risk of an SGA delivery by 76% [28].

We additionally examined whether the above relationships between inter-pregnancy weight change and the risk of adverse pregnancy outcomes were dependent on a women’s baseline BMI at the first pregnancy. In general the risks associated with weight gain (namely gestational hypertension, caesarean delivery and LGA birth) were higher in women who had a healthy BMI (<25) during the first pregnancy than for overweight women, emphasizing that individuals do not necessarily have to become obese *per se* to increase their risk of adverse outcome at the second pregnancy. The exception was the risk of pre-eclampsia which was three-fold higher in women who were overweight at baseline but not different from the weight stable reference group in women who had a healthy weight at this time. This is in sharp contrast to previous data indicating that the effect of inter-pregnancy BMI change on pre-eclampsia was independent of baseline BMI at the first pregnancy [15]. As the approach to the data analysis was identical the reason underlying this discrepancy is unknown but may relate to subtle differences in the diagnosis and hence classification of pre-eclampsia between data bases, or to the relatively higher percentage of women in our population with a large inter-pregnancy BMI gain (3% more in both initially healthy and overweight categories). With respect to those complications associated with inter-pregnancy weight loss, the risk of a SGA birth was independent of BMI at baseline, suggesting that any downward movement between BMI categories is likely to increase the risk of this complication. In contrast the risk of spontaneous preterm delivery following inter-pregnancy weight loss was apparent only in women who had a healthy BMI at baseline. The lack of effect in women who were initially overweight (BMI >25) is in complete agreement with previous work where moving from obese to overweight or normal, and overweight to normal BMI categories did not alter the risk of spontaneous preterm delivery relative to the BMI stable normal reference group [22].

The public health implications of these findings are considerable. The natural assumption is that women who were obese at the start of the first pregnancy are more vulnerable to excessive weight gain during that pregnancy and in the subsequent postpartum period. While this was true for approximately 25% of our study population, a considerable number of initially overweight and obese women (18 and 20%, respectively) lost weight between pregnancies thus theoretically negating the risk of many of the maternal and perinatal complications associated with weight gain but increasing their risk of a SGA birth. In partial support a recent retrospective study involving more than 700,000 women has specifically shown that gestational weight loss protects against pre-eclampsia and emergency caesarean section but increases the risk of prematurity and SGA in all but the most severely obese women [29]. We have no information on the underlying causes of weight change in either direction in the present study but likely candidates not controlled for in the analysis presented here include inappropriate gestational weight change, diet, physical activity, breastfeeding and socio-economic status. Further it is possible that the use of customized birth weight centiles (based on maternal height and weight at booking) to define birth weight extremes may subtly alter the reported relationships. While the consequences of large inter-pregnancy weight gains are arguably more serious for a women’s long term health our results suggest that a degree of caution is required if health professionals promote weight loss at this time, particularly as the effectiveness of dietary and lifestyle intervention strategies aimed at improving pregnancy outcome among both normal and obese women remain largely unproven [30,31].

Together the present and previous studies linking inter-pregnancy BMI change in both directions with a range of contrasting pregnancy complications implies a causal relationship, although we cannot determine whether it is weight change or the behaviours leading to it that confer extra risk. Nevertheless the present study further suggests that some of these relationships may be mediated in part by the placenta. Placental size, morphology, blood flow and nutrient transport functions primarily determine the growth trajectory of the fetus [32] and placental and fetal weight are strongly correlated [33]. Thus in light of the relationship between inter-pregnancy weight loss and SGA (1.76), and between weight gain and LGA ([15] and this study, OR 1.87 and 1.83, respectively), it is perhaps unsurprising that our analysis also reveals that weight loss was associated with a similarly higher risk of low placental weight (OR 1.79), and weight gain with a greater risk of having a large placenta (OR 1.76). The implication is that these changes in maternal nutritional status between successive pregnancies impact maternal nutrient reserves at the start of the second pregnancy and hence the placental growth trajectory. Alterations in placental growth may in turn be on the causative pathway to some of the pregnancy complications studies herein. In partial support, studies in a highly controlled sheep model demonstrate that placental weight is profoundly influenced by BMI at conception (irrespective of subsequent gestational intake) and is closely associated with birth weight at delivery [34]. Moreover in term infants, placental weight has recently been shown to be an important intermediary between maternal conditions of overnutrition (namely pre-pregnancy obesity, excessive gestational weight gain and gestational diabetes mellitus) and increased birth weight [35].

A considerable strength of our study was that weight and height were measured at the first clinic appointment on both occasions and weight was adjusted to a standard gestational age for all maternities. This was a close approximation of pre-pregnancy BMI as it was recorded by clinically trained staff thereby negating recall bias. Moreover the data for the maternal weight corrections was originally derived from women with singleton live births between 32 and 42 weeks gestation from the same geographical area [24], and while arguably sub-optimal for women with stillbirth or post-term delivery is considerably better than no adjustment. Both maternities were at a single hospital and a number of known important confounders were measured and the analyses adjusted accordingly. A further strength is the uniform criteria used in the AMND to record pregnancy complications. On the other hand the study population after exclusions for missing data was relatively small and of low ethnic diversity. Moreover the low event rate for rarer complications such as stillbirth may have limited the power to detect all potential effects. Nevertheless the missing data do not appear to have introduced population bias as the proportion of women per initial BMI category at first pregnancy, the frequency of pregnancy complications in relation to first pregnancy BMI, and the relationship between inter-pregnancy BMI change and the primary incidence of specific pregnancy complications is commensurate with previous publications by other groups using other data sources [5-9,15-22]. Moreover the data was collected over a time range where changes in obstetrical practice might have been expected (eg. criteria for caesarean section) but this potential bias should have been minimized by including year of delivery in the adjusted model. Although the inter-delivery interval was similar between baseline BMI categories, it is acknowledged that the availability of this parameter in years rather than months is not ideal and may have limited the accuracy of adjusting for this parameter in the model.

## Conclusions

In conclusion, inter-pregnancy weight change in either direction variously increases the risk of a number of contrasting pregnancy complications, including extremes of placental weight.

### Consent

This was a retrospective cohort study using routinely collected and fully anonymised data. As such individual patient consent cannot be obtained but a routine opt out clause is applicable at the time of antenatal booking as with all NHS clinical data.

## Competing interests

The authors declare that they have no competing interests.

## Authors’ contributions

JW conceived the study, performed the statistical analysis, interpreted the data and drafted and revised the manuscript. SB and DC are members of the steering committee that manage the AMND and helped critically revise the manuscript. DC also helped conceive aspects of the study. GH provided statistical advice and helped interpret the data, draft and revise the manuscript. All authors read and approved the manuscript.

## Pre-publication history

The pre-publication history for this paper can be accessed here:

http://www.biomedcentral.com/1471-2393/14/40/prepub

## Supplementary Material

Additional file 1 Maternal and pregnancy outcome characteristics at first and second pregnancy in relation to BMI category at first pregnancy. Click here for file

Additional file 2 **Comparison of crude and adjusted odds ratios for all adverse perinatal outcomes during second pregnancy in relation to change in BMI from first pregnancy for women with specific complication in second pregnancy only.** Adjustments as per Table 2 in manuscript. Click here for file

## References

[B1] Lewis G Lewis G The Confidential Enquiry into Maternal and Child Health (CEMACH). Saving mothers’ lives: reviewing maternal deaths to make motherhood safer-2003-2005 The seventh report on confidential enquiries into maternal deaths in the United Kingdom 2007 London: CEMACH http://www.oaa-anaes.ac.uk/assets/_managed/editor/File/Reports/2003-2005_saving_mothers_full_report.pdf

[B2] Ehrenberg HM Intrapartum considerations in prenatal care Semin Perinatol 2011 35 324 329 10.1053/j.semperi.2011.05.016 22108081

[B3] Mamun AA Callaway LK O’Callaghan MJ Williams GM Najman JM Alati R Associations of maternal pre-pregnancy obesity and excess pregnancy weight gains with adverse pregnancy outcomes and length of hospital stay BMC Pregnancy Childbirth 2011 11 62 10.1186/1471-2393-11-62 21892967 PMC3178538

[B4] Bhattacharya S Campbell DM Liston WA Bhattacharya S Effect of body mass index on pregnancy outcomes in nulliparous women delivering singleton babies BMC Public Health 2007 7 168 10.1186/1471-2458-7-168 17650297 PMC1940246

[B5] Salihu HM Lynch O Alio AP Mbah AK Kornosky JL Marty PJ Extreme maternal underweight and feto-infant morbidity outcomes: a population-based study J Matern Fetal Neonatal Med 2009 22 428 434 10.1080/14767050802385764 19530001

[B6] Han Z Mulla S Beyene J Liao G McDonald SD Maternal underweight and the risk of preterm birth and low birth weight: a systematic review and meta-analyses Int J Epidemiol 2011 40 65 101 10.1093/ije/dyq195 21097954

[B7] Manzanares SG Santalla AH Vico IZ Criado LMS Pineda AL Gallo JLV Abnormal maternal body mass index and obstetric and neonatal outcome J Matern Fetal Neonatal Med 2012 25 308 312 10.3109/14767058.2011.575905 21615231

[B8] Abenheim HA Kinch RA Morin L Benjamin A Usher R Effect of prepregnancy body mass index categories on obstetrical and neonatal outcomes Arch Gynecol Obstet 2007 275 39 43 16967276 10.1007/s00404-006-0219-y

[B9] McDonald SD Han Z Mulla S Beyene J Overweight and obesity in mothers and risk of preterm birth and low birth weight infants: systematic review and meta-analyses BMJ 2010 341 c3428 10.1136/bmj.c3428 20647282 PMC2907482

[B10] Dennedy MC Avalos G O’Reilly MW O’Sullivan EP Dunne FP The impact of maternal obesity on gestational outcomes Ir Med J 2012 105 23 25 22838105

[B11] McIntyre HD Gibbons KS Flenady VJ Callaway LK Overweight and obesity in Australian mothers: epidemic or endemic? Med J Aust 2012 196 184 188 10.5694/mja11.11120 22339524

[B12] Bajos N Wellings K Laborde C Moreau C Sexuality and obesity, a gender perspective: results from French national random probability survey of sexual behaviours BMJ 2010 340 c2573 doi: 10.1136/bmj.c2573 10.1136/bmj.c2573 20551118 PMC2886194

[B13] Postlethwaite D Armstrong MA Hung YY Shaber R Pregnancy outcomes by pregnancy intention in a managed care setting Matern Child Health J 2010 14 227 234 10.1007/s10995-009-0446-5 19152103

[B14] Maxson P Miranda ML Pregnancy intention, demographic differences, and psychosocial health J Womens Health (Larchmt) 2011 20 1215 1223 10.1089/jwh.2010.2379 21671765

[B15] Villamor E Cnattingius S Interpregnancy weight change and risk of adverse pregnancy outcomes: a population-based study Lancet 2006 368 1164 1170 10.1016/S0140-6736(06)69473-7 17011943

[B16] Getahun D Ananth CV Peltier MR Salihu HM Scorza WE Changes in prepregnancy body mass index between the first and second pregnancies and risk of large-for-gestational age birth Am J Obstet Gynecol 2007 196 530.e1 530.e8 17547882 10.1016/j.ajog.2006.12.036

[B17] Getahun D Ananth CV Oyelese Y Chavez MR Kirby RS Smulian JC Primary preeclampsia in the second pregnancy: effects of changes in prepregnancy body mass index between pregnancies Obstet Gynecol 2007 110 1319 1325 10.1097/01.AOG.0000292090.40351.30 18055727

[B18] Getahun D Kaminsky LM Elsasser DA Kirby RS Ananth CV Vintzileos AM Changes in prepregnancy body mass index between pregnancies and risk of primary caesarean delivery Am J Obstet Gynecol 2007 197 376.e1 376.e7 17904966 10.1016/j.ajog.2007.06.015

[B19] Whiteman VE Aliyu MH August EM McIntosh C Duan J Alio AP Salihu HM Changes in prepregnancy body mass index between pregnancies and risk of gestational and type 2 diabetes Arch Gynecol Obstet 2011 284 235 240 10.1007/s00404-011-1917-7 21544736

[B20] Whiteman VE Crisan L McIntosh C Alio AP Duan J Marty PJ Salihu HM Interpregnancy body mass index changes and risk of stillbirth Gynecol Obstet Invest 2011 72 192 195 10.1159/000324375 21849757

[B21] Whiteman VE McIntosh C Rao K Mbah AK Salihu HM Interpregnancy BMI change and risk of primary caesarean delivery J Obstet Gynaecol 2011 31 589 593 10.3109/01443615.2011.598968 21973129

[B22] Whiteman VE Rao K Duan J Alio A Marty PJ Salihu HM Changes in prepregnancy body mass index between pregnancies and risk of preterm phenotypes Am J Perinatol 2011 28 67 74 10.1055/s-0030-1262905 20640971

[B23] Wallace JM Horgan GW Bhattacharya S Placental weight and efficiency in relation to maternal body mass index and the risk of pregnancy complications in women delivering singleton babies Placenta 2012 33 611 618 10.1016/j.placenta.2012.05.006 22695104

[B24] Campbell D Hall M Lemon J Carr-Hill R Pritchard C Samphier M Clinical birthweight standards for a total population in the 1980s Br J Obstet Gynaecol 1993 100 436 445 10.1111/j.1471-0528.1993.tb15268.x 8518243

[B25] Tranquilli AL Brown MA Zeeman GG Dekker G Sibai BM The definition of severe and early onset preeclampsia. Statements from the International Society for the Study of Hypertension in Pregnancy (ISSHP) Pregnancy Hypertension: An International Journal of Women’s Cardiovascular Health 2013 3 44 47 10.1016/j.preghy.2012.11.001 26105740

[B26] Bonellie S Chalmers J Gray R Greer I Jarvis S Williams C Centile charts for birthweight for gestational age for Scottish singleton births BMC Pregnancy Childbirth 2008 8 5 10.1186/1471-2393-8-5 18298810 PMC2268653

[B27] Wallace JM Horgan GH Bhattycharya S Gestational age, gender and parity specific centile charts for placental weight for singleton deliveries in Aberdeen, UK Placenta 2013 34 269 274 10.1016/j.placenta.2012.12.007 23332414

[B28] Diouf I Charles MA Thiebaugeorges O Forhan A Kaminski M Heude B Maternal weight change before pregnancy in relation to birth weight and risks of adverse pregnancy outcomes Eur J Epidemiol 2011 26 789 796 10.1007/s10654-011-9599-9 21710259 PMC3925097

[B29] Beyerlein A Schiessl B Lack N von Kries R Associations of gestational weight loss with birth-related outcome: a retrospective cohort study BJOG 2011 118 55 61 10.1111/j.1471-0528.2010.02761.x 21054761

[B30] Tanentsapf I Heitmann BL Adegboye ARA Systematic review of clinical trials on dietary interventions to prevent excessive weight gain during pregnancy among normal weight, overweight and obese women BMC Pregnancy Childbirth 2011 11 81 10.1186/1471-2393-11-81 22029725 PMC3215955

[B31] Oteng-Ntim E Varma R Croker H Poston L Doyle P Lifestyle interventions for overweight and obese pregnant women to improve pregnancy outcome: systematic review and meta-analysis BMC Med 2012 10 47 10.1186/1741-7015-10-47 22574949 PMC3355057

[B32] Wallace JM Mascie-Taylor CGN, Rosetta L Adaptive maternal, placental and fetal responses to nutritional extremes in the pregnant adolescent: lessons from sheep Reproduction and Adaptation 2011 Cambridge: University Press 112 127

[B33] Molteni RA Stys SJ Battaglia FC Relationship of fetal and placental weight in human beings: fetal/placental weight ratios at various gestational ages and birth weight distributions J Reprod Med 1978 21 327 731626

[B34] Wallace JM Milne JS Aitken RP Effect of weight and adiposity at conception and wide variations in gestational dietary intake on pregnancy outcome and early postnatal performance in young adolescent sheep Biol Reprod 2010 82 320 330 10.1095/biolreprod.109.080069 19794151

[B35] Ouyang F Parker M Cerda S Pearson C Fu L Gillman MW Zuckerman B Wang X Placental weight mediates the effects of prenatal factors on fetal growth: the extent differs by preterm status Obesity (Silver Spring) 2013 21 609 620 10.1002/oby.20254 23592670 PMC3418379

