# Supplementary material for: Inter-pregnancy weight change impacts placental weight and is associated with the risk of adverse pregnancy outcomes in the second pregnancy
Source: BMC Pregnancy Childbirth. 2014 Jan 22;14:40. doi: 10.1186/1471-2393-14-40 (PMC3900734; doi:10.1186/1471-2393-14-40)
Supplement: Additional file 1 — Maternal and pregnancy outcome characteristics at first and second pregnancy in relation to BMI category at first pregnancy. [file 1471-2393-14-40-S1.docx]

Additional File 1. Maternal and pregnancy outcome characteristics at first and second pregnancy in relation to BMI category at first pregnancy.

|  | Underweight  BMI < 18.5  n=235 | Normal  BMI 18.6-24.9  n=8031 | Overweight  BMI 25-29.9  n=3342 | Obese  BMI >30  n=1132 | All women  N=12740 |
| --- | --- | --- | --- | --- | --- |
| First pregnancy  Age (years) | 22.8±4.49^a^ | 25.2±4.95^b^ | 26.0±4.75^c^ | 26.1±4.60^c^ | 25.5±4.89 |
| Height (cm) | 162.6±6.48^ab^ | 162.4±6.32^a^ | 161.8±6.30^b^ | 162.2±6.23^ab^ | 162.2±6.31 |
| Weight (kg) | 46.5±4.11^a^ | 57.3±5.95^b^ | 68.6±6.50^c^ | 86.6±11.44^d^ | 62.7±11.31 |
| BMI (kg/m^2^) | 17.6±0.87^a^ | 21.7±1.61^b^ | 26.1±1.47^c^ | 32.9±3.64^d^ | 23.8±3.95 |
| Booking week | 14.2±3.97^a^ | 12.2±3.79^b^ | 11.3±3.67^c^ | 10.7±3.61^d^ | 11.9±3.80 |
| Adjusted weight (kg) | 47.3±4.02^a^ | 59.0±6.13^b^ | 70.6±6.59^c^ | 88.9±11.60^d^ | 64.5±11.57 |
| Adjusted BMI (kg/m^2^) | 17.9±0.62^a^ | 22.3±1.57^b^ | 26.9±1.37^c^ | 33.7±3.65^d^ | 24.5±4.00 |
| Pre-eclampsia | 11 (4.7%)^ab^ | 307 (3.8%)^a^ | 177 (5.3%)^b^ | 119 (10.5%)^c^ | 614 (4.8%) |
| Gestational hypertension | 39 (16.6%)^a^ | 1467 (18.3%)^a^ | 823 (24.6%)^b^ | 351 (31.0%)^c^ | 2680 (21.0%) |
| Induced labour | 59 (25.1%)^ab^ | 2046 (25.5%)^a^ | 1022 (30.6%)^b^ | 472 (41.7%)^c^ | 3599 (28.2%) |
| Instrumental delivery | 50 (21.3%)^a^ | 2207 (27.5%)^b^ | 918 (27.5%)^b^ | 293 (25.9%)^ab^ | 3468 (27.2%) |
| Elective C-Section | 8 (3.4%) | 240 (3.0%) | 121 (3.6%) | 44 (3.9%) | 413 (3.2%) |
| Emergency C-Section | 17 (7.2%)^a^ | 823 (10.2%)^a^ | 568 (17.0%)^b^ | 259 (22.9%)^c^ | 1667 (13.1%) |
| Spontaneous preterm (<37 weeks) | 18 (7.6%) | 567 (7.1%) | 204 (6.1%) | 75 (6.6%) | 864 (6.8%) |
| Post-term delivery (>41weeks) | 9 (3.8%)^ab^ | 461 (5.7%)^a^ | 239 (7.1%)^b^ | 77 (6.8%)^ab^ | 786 (6.2%) |
| Placental abruption | 6 (2.6%) | 119 (1.5%) | 45 (1.4%) | 22 (1.9%) | 192 (1.5%) |
| Placenta praevia | 0 | 11 (0.14%) | 9 (0.23%) | 2 (0.18%) | 22 (0.17%) |
| Postpartum haemorrhage | 23 (9.8%)^ab^ | 836 (10.4 %)^a^ | 291 (8.7%)^b^ | 117 (10.3%)^ab^ | 1267 (9.9%) |
| Stillbirth | 3 (1.28%)^ab^ | 53 (0.66%)^a^ | 29 (0.87%)^a^ | 22 (1.94%)^b^ | 107 (0.84%) |
| SGA | 62 (26.4%)^a^ | 951 (11.8%)^b^ | 274 (8.2%)^c^ | 88 (7.8%)^c^ | 1375 (10.8%) |
| LGA | 8 (3.4%)^a^ | 489 (6.1%)^a^ | 327 (9.8%)^b^ | 161 (14.2%)^c^ | 985 (7.7%) |
| Placental weight <10^th^ centile | 38 (16.2%)^a^ | 848 (10.6%)^b^ | 249 (7.5%)^c^ | 75 (6.6%)^c^ | 1210 (9.5%) |
| Placental weight >90^th^ centile | 13 (5.5%)^a^ | 685 (8.5%)^a^ | 448 (13.4%)^b^ | 201 (17.8%)^c^ | 1347 (10.6%) |
| Second pregnancy  Age (years) | 26.5±4.90^a^ | 28.6±4.96^b^ | 29.3±4.76^c^ | 29.4±4.80^c^ | 28.8±4.91*** |
| Weight (kg) | 49.2±5.89^a^ | 59.3±7.44^b^ | 71.0±9.24^c^ | 89.9±14.04^d^ | 64.9±12.84*** |
| BMI (kg/m^2^) | 18.6±1.80^a^ | 22.5±2.34^b^ | 27.1±2.90^c^ | 34.1±4.82^d^ | 24.6±4.59*** |
| Booking week | 11.8±3.43^a^ | 11.1±3.43^b^ | 10.6±3.31^c^ | 10.1±3.23^d^ | 10.9±3.41*** |
| Adjusted weight (kg) | 51.0±6.14^a^ | 61.5±7.69^b^ | 73.4±9.41^c^ | 92.4±14.22^d^ | 67.1±13.08*** |
| Adjusted BMI (kg/m^2^) | 19.3±1.84^a^ | 23.4±2.37^b^ | 28.0±2.91^c^ | 35.1±4.86^d^ | 25.5±4.64*** |
| Pre-eclampsia | 3 (1.3%)^ab^ | 107 (1.3%)^a^ | 66 (2.0%)^b^ | 49 (4.3%)^c^ | 225 (1.8%)*** |
| Gestational hypertension | 18 (7.7%)^ab^ | 464 (5.8%)^a^ | 321 (9.6%)^b^ | 164 (14.5%)^c^ | 967 (7.6%)*** |
| Induced labour | 39 (16.6%)^ab^ | 1397 (17.4%)^a^ | 668 (20.0%)^b^ | 282 (24.9%)^c^ | 2386 (18.7%)*** |
| Instrumental delivery | 17 (7.2%) | 571 (7.1%) | 244 (7.3%) | 68 (6.0%) | 900 (7.1%)*** |
| Elective C-Section | 12 (5.1%)^a^ | 560 (6.9%)^a^ | 378 (11.3%)^b^ | 157 (13.9%)^c^ | 1107 (8.7%)*** |
| Emergency C-Section | 9 (3.8%)^a^ | 456 (5.7%)^a^ | 276 (8.2%)^b^ | 144 (12.7%)^c^ | 885 (6.9%)*** |
| Spontaneous preterm (<37 weeks) | 27 (11.5%)^a^ | 508 (6.3%)^b^ | 154 (4.6%)^c^ | 56 (4.9%)^bc^ | 745 (5.8%)*** |
| Post-term delivery (>41weeks) | 5 (2.1%) | 243 (3.0%) | 120 (3.6%) | 31 (2.7%) | 399 (3.1%)*** |
| Placental abruption | 1 (0.42%) | 79 (0.98%) | 32 (0.96%) | 17 (1.50%) | 129 (1.01%)*** |
| Placenta praevia | 1 (0.42%) | 34 (0.42%) | 14 (0.42%) | 7 (0.62%) | 56 (0.44%)*** |
| Postpartum haemorrhage | 23 (9.8%)^ab^ | 687 (8.6%)^a^ | 273 (8.2%)^a^ | 122 (10.8%)^b^ | 1105 (8.7)*** |
| Stillbirth | 0 | 25 (0.31%) | 18 (0.53%) | 5 (0.44%) | 48 (0.38%)*** |
| SGA | 48 (20.4%)^a^ | 850 (10.6%)^b^ | 237 (7.1%)^c^ | 68 (6.0%)^c^ | 1203 (9.4%)*** |
| LGA | 6 (2.6%)^a^ | 540 (6.7%)^b^ | 374 (11.2%)^c^ | 173 (15.3%)^d^ | 1093 (8.6%)** |
| Placental weight <10^th^ centile | 43 (18.3%)^a^ | 707 (8.8%)^b^ | 198 (5.9%)^c^ | 51 (4.5%)^c^ | 999 (7.8%)*** |
| Placental weight >90^th^ centile | 13 (5.5%)^a^ | 713 (8.9%)^a^ | 472 (14.1%)^b^ | 231 (20.4%)^c^ | 1429 (11.2%) |
| Inter-delivery interval (years) | 3.7±4.90^a^ | 3.4±4.96^a^ | 3.3±4.76^b^ | 3.3±4.79^ab^ |  |

Values are mean (SD) or number (per cent). Univariate analysis was conducted using one-way ANOVA for continuous variables and where significant effects were detected, individual maternal BMI categories were subsequently compared by Tukey’s method. Chi-Square tests were used to test independence among all possible BMI group comparisons for categorical variables. Within rows any means which do not have a superscript in common are significantly different, and otherwise are not, minimum P<0.05. The overall population characteristics at first versus second pregnancy were compared by paired Students t test for continuous variables and by McNemar’s test for categorical variables, **P<0.01, ***P<0.001.
