# Supplementary material for: Inter-pregnancy weight change impacts placental weight and is associated with the risk of adverse pregnancy outcomes in the second pregnancy
Source: BMC Pregnancy Childbirth. 2014 Jan 22;14:40. doi: 10.1186/1471-2393-14-40 (PMC3900734; doi:10.1186/1471-2393-14-40)
Supplement: Additional file 2 — Comparison of crude and adjusted odds ratios for all adverse perinatal outcomes during second pregnancy in relation to change in BMI from first pregnancy for women with specific complication in second pregnancy only. Adjustments as per Table 2 in manuscript. [file 1471-2393-14-40-S2.docx]

Additional File 2. Comparison of crude and adjusted odds ratios for all adverse perinatal outcomes during second pregnancy in relation to change in BMI from first pregnancy for women with specific complication in second pregnancy only. Adjustments as per Table 2 in manuscript

|  |  | BMI Change (% study population) | | | |  | |  |
| --- | --- | --- | --- | --- | --- | --- | --- | --- |
| Complication in 2^nd^ pregnancy only (cases) |  | <-1†  (13%) | -1 to <1  (44%) | 1 to <3  (29%) | >3  (14%) | | P value | |
| Pre-eclampsia  (n=140) | CrudeOR(95%CI)  Adj OR(95% CI) | 1.50 (0.86-2.62)  1.23 (0.69-2.20) | 1  1 | **1.65 (1.08-2.52)***  1.44 (0.93-2.23) | **2.80(1.78-4.39)*****  **1.85 (1.12-3.04)*** | | **<0.001**  0.100 | |
| Gestational hypertension n=524) | CrudeOR(95%CI)  Adj OR(95% CI) | 1.00 (0.74-1.35)  0.83 (0.61-1.14) | 1  1 | **1.26 (1.01-1.56)***  1.22 (0.98-1.52) | **1.96 (1.55-2.49)*****  **1.82 (1.40-2.36)***** | | **<0. 001**  **<0.001** | |
| Induced labour (n=1371) | CrudeOR(95%CI)  Adj OR(95% CI) | **1.23 (1.03-1.46)***  **1.21 (1.01-1.46)*** | 1  1 | **1.17 (1.02-1.34)***  1.12 (0.98-1.29) | **1.35 (1.14-1.59)*****  1.17 (0.97-1.40) | | **0.002**  0.100 | |
| Elective caesarean  (n=930) | CrudeOR(95%CI)  Adj OR(95% CI) | 1.05 (0.84-1.30)  0.91 (0.72-1.15) | 1  1 | **1.22 (1.04-1.43)***  1.16 (0.98-1.37) | **1.38 (1.14-1.68)****  1.18 (0.95-1.48) | | **0.005**  0.093 | |
| Emergency caesarean(n=461) | CrudeOR(95%CI)  Adj OR(95% CI) | **1.37 (1.01-1.87)***  1.27 (0.93-1.75) | 1  1 | **1.44 (1.14-1.82)****  **1.30 (1.03-1.66)*** | **2.50 (1.94-3.21)*****  **1.78 (1.35-2.35)***** | | **<0. 001**  **<0.001** | |
| Spontaneous preterm (<37wks, n=558) | CrudeOR(95%CI)  Adj OR(95% CI) | **1.34 (1.05-1.72)***  **1.46 (1.08-1.97)*** | 1  1 | 1.03 (0.84-1.26)  1.00 (0.78-1.28) | 0.93 (0.71-1.23)  **0.65 (0.45-0.93)*** | | 0.098  **0.002** | |
| SGA, <10^th^ C (n=800) | CrudeOR(95%CI)  Adj OR(95% CI) | **1.49(1.22-1.83)*****  **1.65(1.33-2.04)***** | 1  1 | 0.98 (0.83-1.17)  0.95 (0.80-1.14) | 0.91 (0.72-1.15)  0.82 (0.63-1.05) | | **<0.001**  **<0.001** | |
| LGA, >90^th^ C (n=755) | CrudeOR(95%CI)  Adj OR(95% CI) | **0.67 (0.50-0.89)****  **0.57(0.42-0.76)***** | 1  1 | **1.49(1.25-1.77)*****  **1.48(1.24-1.76)***** | **1.81(1.47-2.22)*****  **1.70 (1.36-2.13)***** | | **<0. 001**  **<0.001** | |
| Placental Wt. <10thC (n=773) | CrudeOR(95%CI)  Adj OR(95% CI) | **1.39(1.13-1.71)****  **1.67(1.35-2.07)***** | 1  1 | 0.89 (0.75-1.07)  0.91 (0.76-1.09) | 0.87 (0.69-1.10)  0.99 (0.77-1.28) | | **0. 001**  **<0.001** | |
| Placental Wt. >90thC (n=1061) | CrudeOR(95%CI)  Adj OR(95% CI) | 1.06 (0.85-1.31)  0.90 (0.72-1.11) | 1  1 | **1.31 (1.12-1.53)****  **1.26 (1.08-1.47)**** | **1.87 (1.57-2.23)*****  **1.59 (1.31-1.47)***** | | **<0. 001**  **<0.001** | |
| Stillbirth  (n=46) | CrudeOR(95%CI)  Adj OR(95% CI) | 1.82 (0.77-4.31)  1.43 (0.54-3.77) | 1  1 | 1.60 (0.79-3.23)  1.50 (0.71-3.18) | 1.47 (0.60-3.61)  0.74 (0.26-2.12) | | 0.469  0.434 | |
| Post-term delivery (>41 wks, n=302) | CrudeOR(95%CI)  Adj OR(95% CI) | 0.79 (0.54-1.17)  1.26 (0.53-3.02) | 1  1 | 0.95 (0.72-1.24)  1.49 (0.84-2.62) | 1.06 (0.75-1.48)  1.64 (0.74-3.64) | | 0.620  0.444 | |
| Postpartum haemorrhage(n=887) | CrudeOR(95%CI)  Adj OR(95% CI) | 1.17 (0.94-1.45)  1.06 (0.85-1.33) | 1  1 | **1.18 (1.01-1.39)***  1.07 (0.90-1.26) | **1.30 (1.06-1.59)***  0.88 (0.70-1.10) | | **0.047**  0.336 | |
| Placental abruption (n=115) | CrudeOR(95%CI)  Adj OR(95% CI) | 1.33 (0.75-2.39)  1.17 (0.63-2.20) | 1  1 | 1.17 (0.73-1.86)  1.03 (0.63-1.68) | **2.01 (1.23-3.29)****  1.17 (0.66-2.07) | | **0.047**  0.933 | |
| Placenta praevia (n=56) | CrudeOR(95%CI)  Adj OR(95% CI) | 0.59 (0.20-1.72)  0.56 (0.19-1.68) | 1  1 | 1.17 (0.63-2.17)  1.10 (0.58-2.07) | 1.51 (0.73-3.10)  1.21 (0.54-2.72) | | 0.410  0.633 | |
